# A’uwẽ (Xavante) views of food security in a context of monetarization of an indigenous economy in Central Brazil

**DOI:** 10.1371/journal.pone.0264525

**Published:** 2022-02-25

**Authors:** James R. Welch, Carlos E. A. Coimbra

**Affiliations:** Escola Nacional de Saúde Pública, Fundação Oswaldo Cruz, Rio de Janeiro, Rio de Janeiro, Brazil; North Carolina State University, UNITED STATES

## Abstract

Following boom-and-bust economic cycles provoked by Brazilian governmental attempts to integrate Indigenous peoples into national society, it is approximately since the beginning of the 2000s that Brazilian Indigenous peoples came to be viewed officially as “poor” and victims of “hunger.” Consequently, the national indigenist agency and other State entities started to conceive and implement diverse initiatives that ultimately injected money and resources into Indigenous communities. In 2019 we undertook an ethnographic study in three A’uwẽ (Xavante) communities in the Pimentel Barbosa Indigenous Reserve, Central Brazil, with the objective of analyzing how people understand and pursue food security. We propose that in the studied communities the complex network of A’uwẽ food reciprocity is a fundamental strategy for mitigating hunger and acute lack of food. We show that among the A’uwẽ, the hybrid economy that developed since the 1970s has proved resilient to dramatic transformations and uncertainty in the availability and characteristics of external government inputs.

## Introduction

Historically, hunger, food insecurity, food production, and economic self-sufficiency have been recurrent themes in Brazilian indigenist politics, associated with the colonial process of “pacification,” which involves the attraction and fixation of an Indigenous group to an indigenist post or religious mission and, subsequently, opening of the greater part of their original territories for the implementation of developmentalist projects [1, 2]. This policy, based in now antiquated notions of acculturation, was a primary orienting principle of the Brazilian indigenist agency (the Indian Protection Service [SPI] followed by the National Indian Foundation [FUNAI]), which always tended to the side of the government aspirations to develop and “integrate” Indigenous peoples into Brazilian market-oriented society [2–5]. In seeking to make Indigenous people economically self-sufficient, the government intervened in Indigenous economies by means of top-down agricultural (coffee, rice, sugarcane) and extractivist (natural rubber, Brazil nut) community projects. This boom-and-bust governmental approach to Indigenous people’s economies and food security prevailed throughout the twentieth century and continues in different forms in present times.

It is approximately since the beginning of the 2000s that Brazilian Indigenous peoples came to be viewed officially as “poor” and victims of “hunger.” Consequently, FUNAI and other State entities, including the Judiciary Power, started to conceive and implement diverse initiatives that ultimately inject money and resources into communities by means of salaries, retirement pensions, distribution of basic food baskets (*cestas básicas de alimentos*), school meal programs, maternity financial assistance (*salário maternidade*), and the cash transfer program known throughout the country as Bolsa Família (Family Allowance) [6].

A major flaw in the vast majority of these policies and programs, especially those involving cash transfers, is that they do not plan for periodic evaluative research in the field. The Bolsa Família Program, considered the largest cash transfer program in the world, has as its primary objective the reduction of “poverty” in Brazil; only indirectly or secondarily addressing a number of objectives related to “human development”, including food and nutritional security as well as schooling and healthcare [6]. Therefore, it is not surprising that studies carried out with Indigenous and riverine populations in the Amazon show that receipt of Bolsa Família by families was associated with lower food security, and did not improve the nutritional status of benefited children [7, 8].

Other recent studies question the effects of these social programs, arguing that the Indigenous beneficiaries act with agency in reinterpreting the uses and destinations of benefits, particularly financial. The Sateré-Mawé case in Amazonas state is particularly emblematic of the economic contradictions arising from governmental programs conceived without any Indigenous participation, with adverse impacts on subsistence and, consequently, the population’s food security [9]. Also in Amazonas, among the Indigenous peoples of the Upper Rio Negro region, Athila [10] emphasizes the unexpected social, economic, and health implications brought about by the Bolsa Família program and other public policies that imposed new needs to travel between communities and city, not only for people to withdraw their money from banks, but also to go shopping in local stores.

From a nutritional standpoint, data from the First National Survey of Indigenous Peoples’ Health and Nutrition in Brazil as well as from selected regional surveys, confirm the magnitude of unacceptable health disparities these people endure, with high prevalence of food insecurity and undernutrition coexisting with obesity and early-onset of chronic metabolic disorders [11–15].

We undertook an ethnographic study in three A’uwẽ (Xavante) communities in the Pimentel Barbosa Indigenous Reserve, Central Brazil, with the objective of ascertaining how people perceive public policies of social inclusion, food access, sharing, and hunger. In this paper, we discuss our findings to elucidate relationships between Indigenous agency, social organization, and public policies.

Perceptions of food security are cultural, situational, and individual. In this paper, we will focus on the first two, where the main cultural aspect is reciprocity and food sharing between family members and neighbors; the situational aspect is linked to the increasing monetarization of domestic economies through government programs. Based on an ethnographically informed analysis, we propose that in the studied communities, governmental social programs, pensions, and salaries were salient resources for accessing basic food supplies on a routine basis, but not during times of hunger. Rather, acute lack of food was more commonly addressed through the complex network of A’uwẽ food reciprocity.

## Methods

The present study is part of a larger research project coordinated by Ricardo Ventura Santos that addresses Indigenous agency in relation to public health policy and information systems [16, 17]. Fieldwork was carried out in Pimentel Barbosa, Etênhiritipá, and Novo Paraíso communities by both authors with eleven A’uwẽ translators and two non-A’uwẽ field assistants in June and July 2019. Ethnographic research included household visits and interviews with all available adult participants who responded to our invitation. Adulthood was defined according to A’uwẽ cultural convention as women and men who had participated in rites of initiation into novitiate adulthood (*danhono*) or women who were married. No sampling methodology was employed.

This study was performed in line with the principles of the 1964 Declaration of Helsinki and amendments. Approval was granted by the Brazilian National Research Ethics Commission (CAAE 61230416.6.0000.5240). Permission to conduct research in the Pimentel Barbosa Indigenous Reserve was granted by FUNAI (N° 59/AAEP/PRES/2019).

Terms of informed consent were presented in the culturally appropriate public community forums, evening council meetings in each community. Per Brazilian law (National Health Council resolutions 196/96 and 304/00) and approval of our consent procedures by the National Research Ethics Commission, community approval was registered on a Collective Free and Informed Consent Form signed by recognized leaders (usually chiefs and vice-chiefs). Invitation to participate was communicated in the evening council meetings, as well as during visits to each household. Interested interviewees were requested to indicate consent orally and freely permitted to not participate or withdraw from participation at any time without prejudice.

We chose to conduct open interviews addressing social policies, food access and sharing, and hunger. Psychometric scales of food insecurity, especially the Brazilian Food Insecurity Scale, were not applied due to limitations in the comparability of their results after necessary adaptation for socioculturally distinct Indigenous peoples. The Brazilian scale, amply utilized in studies of the country’s non-Indigenous population, has proved difficult to generalize among its approximately 300 Indigenous ethnic groups and has contributed little to understanding the concept of “food insecurity” within studied populations [18]. Given the complexity of the diverse food contexts experienced by each Indigenous people, we prioritized an ethnographic approach which, as exemplified by other authors, provided greater potential to explore more deeply central themes of relevance to the A’uwẽ people and their dynamic food economy, as well as to relate these to broader processes of monetarization of Indigenous economies throughout the country [see 18, 19].

Interviews were conducted at volunteer interviewee’s households in Portuguese with the assistance of A’uwẽ translators when preferred by interviewees and recorded on portable audio devices. Interviewees were oriented to address the guiding questions: (1) Describe if you benefit from social policies, how they affect your access to foods, and your general opinion of their usefulness; (2) Describe your overall access to food and the role of sharing with other households; and (3) Describe your experiences of hunger or fearing hunger and how you alleviate such circumstances. Interviewees were allowed to respond to these open questions without interruption except in cases where light redirection was necessary to stimulate communicativeness or refocus attention on research topics. Both authors independently perceived through their personal participation as interviewers that response saturation regarding key questions was achieved well before all interviewed were completed.

Recordings of interviews were transferred from audio recording devices to a password-protected research computer with two-factor authentication cloud-based backup. Subsequently, interviews were transcribed to facilitate ethnographic analysis, with transcription files being securely stored with interview recordings for possible future analysis of topics related to the goals of the umbrella project. Similarity among messages, meaningful examples, and relevant exceptions were identified and interpreted by the authors based on their ethnographic readings of the interviews and deep familiarity with this A’uwẽ population (18 and 32 years for the authors, respectively).

## Study population

The three study communities are all historically derived from the same population that crossed westward over the das Mortes River in the early 1970s and established itself close to the recently created FUNAI post, also called Pimentel Barbosa [20]. The first community, also denominated Pimentel Barbosa, has one of the largest populations in the reserve, totaling 471 people at the time of our fieldwork in 2019. In 2006, a group departed from Pimentel Barbosa community and settled less than one kilometer away. This community is called Etênhiritipá, with a total of 338 people. The third community included in the study, called Novo Paraíso and established in 2013, also began as a faction that left Pimentel Barbosa community. This community had a population of 113 individuals.

In the 1950s, when anthropologist David Maybury-Lewis [21] undertook his ethnographic study among the A’uwẽ who today live in the Pimentel Barbosa Indigenous Reserve, and with whom our present study also was done, agriculture was viewed as a secondary activity to hunting and collecting wild tubers and roots. Between the time Maybury-Lewis conducted his research and anthropologist Nancy M. Flowers initiated her studies in the same community 20 years later, important economic, political, and environmental changes occurred in eastern Mato Grosso, with notable impacts for A’uwẽ society [22].

In the 1970s and 1980s, discourse of promoting integration of Indigenous peoples into the national economy, especially ethnic groups located in regions of the country considered “demographically empty” or “underdeveloped,” was accompanied by injection of large sums of governmental resources to stimulate mechanized agriculture [20, 23, 24]. The A’uwẽ were in the crosshairs of the government’s developmental policy, seen for their potential as rural labor to transform Indigenous lands into large producers of grains and cattle in the cerrado, a tropical savannah landscape typical of Central Brazil. As has been the case since its inception, FUNAI was completely in line with governmental developmental policy [2, 3].

Concretely, FUNAI inaugurated a mechanized rice-growing project in A’uwẽ lands, aimed at producing surpluses to be sold in the regional market. Best known as the “Xavante Project,” this endeavor caused great euphoria in the communities. Several anthropologists who conducted research among the A’uwẽ in the 1970s and 1980s were uniformly impacted by the Xavante Project, highlighting its “grandiosity” [25] and “elevated investments” [26]. According to Maybury-Lewis, who visited A’uwẽ lands at the apex of the project, “The Shavante Project is undoubtedly the most ambitious development project which has been undertaken in recent years by FUNAI on behalf of any single group of Indians” [27].

Despite the initial enthusiasm, the Xavante Project quickly dwindled and failed, as did so many other similar initiatives. The generous initial investments were not continued and the agricultural machines and trucks, without adequate maintenance and without sufficient funds to guarantee replacement parts and even fuel, were rapidly abandoned in the cerrado, left in place where they broke.

Anthropologists who conducted research during the rice project were influenced by the prevailing view in the anthropology of the 1960s and 1970s, according to which changes taking place in Indigenous societies and economies in the Amazon were unidirectional, culminating in the “integration” of Indigenous peoples into the market economy. In the case of A’uwẽ, this process was to occur by means of mechanized rice production [28, 29]. Approximately two decades later, upon returning to the A’uwẽ of Pimentel Barbosa for a restudy, it was observed that this prediction was not confirmed. To the contrary, we showed that beginning in the 1990s, after the failure of the FUNAI rice project, the A’uwẽ did not continue to follow the path of continued agricultural intensification. Quite differently, they began dedicating less time to agriculture and more to the procurement of wild resources inside their reserve, with emphasis on hunting, fishing, and collecting fruits and tubers [23, 30].

What, then, remained for the A’uwẽ of this indigenist agro-industrial experiment? Foremost is the rice, which today comprises an overwhelmingly important part of the daily diet in practically all communities. Grown in traditional swidden gardens, bought in the local markets, or received through basic food baskets or other donations, rice has become a quick and easy substitute for the many traditional varieties of roots and tubers. From the nutritional point of view, the introduction of rice as the dietary staple was deleterious for the A’uwẽ because it is a main trigger of the accelerated nutritional transition underway in practically all communities and the fast-growing prevalence of obesity and diabetes mellitus type II in most communities [13, 23, 31–33].

Another important inheritance of the Xavante Project is the emergence of monetarization of domestic and community economies. It was during this rice project that the first A’uwẽ began to receive salaries, in general paid by FUNAI. Young A’uwẽ were contracted for positions as tractor operators, truck drivers, and among other jobs necessary for the functioning of the agricultural project. Even after the end of the project, many A’uwẽ maintained their public service employment and continue to receive salaries or pensions today. At the turn of the 1960s to the 1970s, some young people who were unable to get jobs with FUNAI accepted job offers from neighboring farmers. During this period there was a great scarcity of rural labor in the region, and the A’uwẽ attended, at least in part, the demand for temporary work on the ranches, principally during the season of harvesting and drying rice. Whether via FUNAI or ranchers, money came to circulate in the A’uwẽ communities during the rice project and, therefore, it is since the 1970s that A’uwẽ society was effectively monetarized.

Many transformations have occurred since the 1970s. FUNAI, which had already undergone successive political crises since the mid-1980s and faced strong tension in its dealings with the A’uwẽ, completely abandoned the Pimentel Barbosa post at the end of the 1990s. Currently, the former post’s location is now occupied by a recently constructed health post with staff accommodations, attending to Pimentel Barbosa and Etênhiritipá communities. Adjacent to the health post is a municipal elementary schoolhouse for Pimentel Barbosa community. Etênhiritipá is served by a state schoolhouse located within their nearby community.

The health post offers at least two paid jobs (Indigenous health agent and Indigenous sanitation agent) for A’uwẽ residents of each of two communities served. Presently, the educational system also contributes to community economies where schools are present, such as Pimentel Barbosa and Etênhiritipá. The elementary schools established in these communities follow the pattern of Brazilian rural schools, with numerous employment positions for teachers of different grades, as well as a full administrative staff. All school employees are A’uwẽ people living in the community served, which results in a significant entry of salaries into the budgets of households with employees and indirectly into extended families and whole communities. These health and education employment positions are formally instituted according to legislation entitling employees to vacation time and a thirteenth monthly salary each year.

If, on the one hand, Pimentel Barbosa and Etênhiritipá communities receive health and elementary school services with significant work opportunities for their residents, the more recently established Novo Paraíso community does not receive either kind of service. Consequently, there are no jobs available for young people, which is cause for continued pressure on leaders and government agencies. The community has only one retired Indigenous FUNAI employee remaining from the time of the extinct rice project. Accordingly, most households in this community rely exclusively on social benefits for monetary income.

Although the A’uwẽ communities of Novo Paraíso, Etênhiritipá, and Pimentel Barbosa are located at different distances by road from centers of commerce, all engage in what has been called a “hybrid economy” [34, 35], involving Indigenous food production, market participation, and government inputs. Residents of all communities have direct or indirect access to financial resources to purchase foods and continue to benefit from garden and cerrado food resources. Furthermore, different households have unequal direct access to garden, cerrado, and market foods associated with an emergent process of socioeconomic differentiation inside A’uwẽ communities [23, 32]. These differences predispose some households to depend to greater or lesser degrees on Indigenous production, including gardening, collecting, fishing, and hunting. Households with greater access to monetary resources have more capacity to purchase food and therefore more options regarding their degree of investment in food subsistence activities. Also, some people have contrastive preferences unrelated to income availability as to whether to invest in these activities. In this kind of mixed food economy, differences between households deserve attention to avoid overgeneralization about how members of the same ethnic group or community provide food for their domestic groups.

Market foods are purchased in three types of commercial outlets: regional market centers, roadside restaurants and stores, and traveling automobile vendors. Many people travel to the main regional market centers, including the towns of Água Boa, Canarana, and Riberão Cascalheira, at least once a month to collect their income at banks and purchase food, clothes, and other necessities. Food is usually purchased at supermarkets, often in big containers for such staples as rice and beans. Clothing and sundry items are often purchased in stores catering to the A’uwẽ and the Indigenous population from the Upper Xingu River.

## Results

The overall study population included 743 adults, of which 389 (52.4%) were women. Members of 5 households were absent at the time of the study (4 women and 5 men, representing 1.1% and 1.4% of the totals, respectively). Invitation to participate in open interviews was accepted by 39 people (5.3% of the available adult population), of which 19 (48.7%) were women. The age distribution of interviewees was 8 (20.5%) from 14 to 24 years, 13 (33.3%) from 25 to 34 years, 6 (15.4%) from 35 to 44 years, and 12 (30.8%) over 45 years.

Ethnographic interviews suggested that most individuals were little satisfied with the food in their households. Feeling hungry and worrying about food running out were prevalent themes, whereas going without food altogether was not. Another recurrent theme was that many members of younger generations no longer appreciate or were not interested in traditional or locally produced A’uwẽ foods. Many interviewees, both young and old, highlighted the preference of younger individuals to eat food purchased in the city. Many young mothers reported exclusively purchasing food in town rather than participating in local subsistence activities to produce or collect food within the Indigenous reserve. For example, according to one elder woman:

“*[…] only I eat A’uwẽ foods because the grandchildren are entering the life of the ‘whites’ [*…*] older people ate A’uwẽ food*, *but today the young generation is entering the life of the ‘whites’ [*…*] sometimes the price is very expensive*.*”*

From the perspective of a young mother, “for food on a daily basis, I shop in the city, right, it’s not here in the Indigenous land, but I buy sometimes in the city…”. Another young mother reinforces the pattern: “[…] I only buy in the city, I’m the new generation, you know, I don’t produce on Indigenous land now. Now I buy food out there”. A young man expresses this pattern in terms of personal taste:

“*I’m really enjoying the city food*, *I’m really enjoying it*. *I’m enjoying everything very much*. *I don’t like A’uwẽ food*, *I don’t*. *We young people do not like A’uwẽ food*, *we are only buying to eat in the city*.*”*

Older adults also report being accustomed to buying food and goods, as one elder man reported:

“*So*, *I live on my salary*. *We no longer live as we did long ago*, *when we didn’t have this money*, *didn’t have the things of the ‘whites’*. *Now the things of the ‘whites’ are arriving*, *which we give lots of value*, *lots of importance*. *Even me*, *when I run out of money*, *I get discouraged*, *right*?*”*

The preference for market foods is maintained despite many individuals observing that in their opinions, purchased foods bring diseases that are avoided by a traditional diet. One man explained, “white people’s food is good, but it brings disease, many diseases”.

Both men and women reported managing the financial resources of the household, suggesting that there is no fixed pattern among the A’uwẽ. According to the diversity of responses, who manages financial resources depends on the household composition, the ability of each earner to plan and budget spending in Brazilian currency, and the perception of what managing financial resources entails. According to an elderly mother:

“*I’m the one who decides*, *because only I have a retirement pension here*. *I do the shopping*. *Only I take care of the house and decide what to buy for the house*. *I use my own money for my home*. *I run the family*.*”*

A younger mother emphasized the important role women play in managing household financial resources, “I decide to buy clothes for the children. Here men don’t run things, it is the woman who decides.” A younger father characterizes financial management in his household as a collaboration between himself and his two wives:

“*I have two wives*. *So*, *before buying*, *we discuss before leaving for the city*. *So*, *we discuss what we are going to buy for our house*. *Arriving in the city*, *everything is already planned as to what to buy*.*”*

Yet another young father explains how he purchases the food, but the women of the household tell him how to spend it, “I receive R$180 from Bolsa Família. I spend this money buying food for the family, but who decides are the women.” Similar statements were recurrent in our interviews.

These quotations reflect cultural patterns surrounding the themes of individual and collective property, as well as the roles of women versus men in the decisions made regarding spending choices. Traditionally, when women collect food, they do so on their own without input from their husbands. When men hunt or fish, they also do so according to their own decisions, without interference from their wives. Spending money seems to occupy an intermediate position between these two cultural models, since the decision of what to buy may be retained by women even when men do the shopping. A male community leader commented on this dynamic for his household:

“*We men work hunting outside the village*, *but when we arrive at home with the game meat*, *it is the woman who distributes the meat*. *Inside the house*, *it is the woman who resolves these things*. *[…] the money I receive I don’t spend for myself*, *right*? *My necessities are not just for me*, *so*, *if the children want some little thing*, *we have to attend to our children*. *If the wife also wants something*, *clothing*, *and I have the money*, *I have to attend to her*. *[…] I can’t spend like that*, *as I would wish*, *sometimes I have to consult my wife to attend to the desires of the family*, *the children*. *Each one has his or her own desires*.*”*

Decisions to share food with other households appears to be the exclusive domain of women. An older woman explained how she distributes purchased and garden foods to her sons’ households as well as houses in other communities:

“*[*…*] I am the only mother here*, *my children have a home*. *On a daily basis I buy and distribute to the children*, *I share*. *Here in the village my husband has a garden*, *he has bananas*, *so he harvests and I distribute to others in the Indigenous land*.*”*

Her son provided a complementary statement, explaining that because he earns very little money, the food in his house disappears quickly. In these moments, his mother helps, as she does for her other sons. He also mentioned that at other times, when he has the money to purchase food, it is shared with other houses to help them.

One young woman reported that the decision to share food was hers alone, as well as the decision to ask for food from other households when hers had ran out. Her discourse is punctuated with the unhappy observation that there are many mouths to feed, such as her father-in-law who eats at her house, leaving her very little satisfied with the food in the household. From the perspective of the person asking for food, one young father reported:

“*when food runs out at my house*, *I borrow from someone close to the family or a neighbor […] my wife or I go to my mother’s house or the house of another person in the family*. *We borrow food on loan*, *waiting for the day when we will receive money*. *If the village car isn’t available*, *broken down*, *then it will take time to get food*, *so then*, *whenever food is missing*, *we ask the family and the neighbor*.*”*

One woman characterized the new custom of eating only market foods, rather than replenishable traditional foods, as connected to the necessity of routinely asking for food from other households:

“*[…] as the young generation isn’t accustomed to eating A’uwẽ food*, *it is difficult for me to sustain food […] but every family has some house where they can ask for a little beans*, *some rice*, *or things like that for the family*, *because the youth are not accustomed to A’uwẽ food*.*”*

Similarly, an elder man reported that in his evaluation, worrying about a lack of food, or feeling a lack of food can be attributed to increased reliance on market foods:

“*As for the food in my house*, *I am satisfied*. *This despite being worried at the end of the month*, *because everything starts to run out*. *The ‘whites’ taught us all the things that make us suffer […] before*, *I didn’t miss city food*, *before we ate fish*, *game*, *didn’t worry about anything*. *Now*, *we have to buy things from the market*, *like rice and beans*, *soy oil […] now we buy these things*, *but there’s not enough money*.*”*

Satisfaction with one’s household food conditions was often attributed to the happiness of one’s children when food is plentiful. For example, one man reported that “I am satisfied when there is a lot of food at home, the children are happy with the food, they are going to celebrate, play, that’s when I am very satisfied.”

Dissatisfaction with one’s household’s food was also a common theme, often mixed with comments or complaints about how certain relatives deplete food reserves by eating at their houses. A recurrent statement was that immediately after shopping in the market, when the house was full of food, people were content with their household food situation. However, after relatives visited and ate their food or took food as gifts, the reserves became depleted and people became dissatisfied with their household food conditions. For example, according to one young woman:

“*Whenever my husband receives his salary*, *he brings food and then I am very happy*. *Then my husband’s brother comes to visit and takes something to feed his family*. *So I get sad when my husband gives something to his brother because the food in our house becomes scarce*.*”*

Similarly, according to another young woman:

“*It’s tough*, *right*? *I go to the city and come back with food*, *but neighbors and relatives come to my house and I give them something*. *I earn little money for shopping*, *so if someone comes to my house where I have little*, *I buy very little*, *I can only distribute a little food to people who come to ask*. *Food is complicated*!*”*

The theme of satisfaction upon purchasing foods and dissatisfaction upon their distribution to relatives and neighbors and consequent depletion was reported by other young women and demonstrates that reciprocity and food sharing is not without problems. We were aware of an acute ambiguity between the importance for well-being (hunger mitigation) of asking or receiving food from others and the dissatisfaction that resulted from depleting food resources by sharing and attending to requests.

Another interview illustrates a pattern of worrying about how to feed children when food runs out:

“*When I have a lot of food in the house*, *I am satisfied because my family celebrates*. *However*, *if it runs out*, *it becomes difficult for me*: *how will I support the family*?*”*

According to some interviewees, when food runs out at home, they look to traditional subsistence activities to meet the households demands. This was a recurrent but less common response. For example, according to a young mother, “when the food runs out, I’ll go to the cerrado to get traditional food to sustain the children.” Similarly, a young man described hunting with his elders when the market food ran out, and sharing the resulting game meat with others:

“*On a daily basis I eat from the city*, *but here the food runs out*. *So*, *I go out hunting with the elders and bring more food to the house*. *It’s good to get Bolsa Família*, *but it runs out*.*”*

These final reports show that reciprocity is the principal but not the only hunger mitigation strategy for households among the A’uwẽ.

## Discussion and conclusion

Cited interviews illustrate several key patterns related to how people understood and mitigated hunger and acute lack of food according to their own logics and strategies. Our results do not highlight public policies aimed at mitigating food insecurity because these were not prominent in A’uwẽ discussions of public policy and food security. Rather, these programs were most commonly identified as meager resources for routine (monthly) food acquisition. For example, although children in a vast majority of households benefit from school meal programs, these were rarely mentioned by interviewees as an important factor in their perceptions of social programs, access to food, and hunger. Other programs, such as the national Food Acquisition Program (Programa de Aquisição de Alimentos), which was implemented in 2003 with the goal of encouraging family farming and reducing food insecurity, did not benefit any households in the study communities.

An initial observation was that many people, especially members of younger generations, now prefer market to traditional foods, just as many households have come to rely almost exclusively on purchased foods rather than traditional foods, despite increased cost and potential deleterious health consequences. Our data do not permit a conclusive or exhaustive explanation of this pattern, although some participants attributed this change to personal preference, becoming accustomed to diverse industrial and processed foods, greater access to financial resources, more frequent and convenient purchasing opportunities, or dislike of physically demanding self-provisioning activities. We further speculate that people, especially younger people, now prefer market foods because of complex and interrelated dimensions of several historically recent transformations including sedentarization of people and communities, monetarization of community economies, household connection to the electrical grid, and more pervasive exposure to culturally Brazilian information, ideas, values, aesthetics, and identities.

Interviewees reported diverse decision-making configurations and processes related to money and food management, but, most frequently, women were reported to make decisions as to how money would be spent on food and controlled how food would be managed and shared once it arrived at home. Sharing or loaning food was a universal theme, but not free from ambiguous feelings. Nearly every interviewee reported sharing food as a provider or receiver, but many female food managers reported dissatisfaction with the resulting depletion of food resources by assisting relatives and neighbors. These two themes, food insecurity resulting from cultural transformations accompanying insertion into Brazilian society and market economy and the persistence of mitigating food reciprocity traditions, were identified by an A’uwẽ scholar as key factors affecting contemporary food culture in the São Marcos Indigenous Land to the south of Pimentel Barbosa [36].

Food reciprocity was frequently emphasized for market foods, in addition to traditional or garden foods. Some individuals observed that reciprocity had increased due to reliance on market foods, because their scarcity led to the need to ask for loans or gifts more frequently. Similarly, some individuals attributed worrying about a lack of food or feeling hungry to increased reliance on market foods. Food satisfaction was often described in terms of children’s happiness with an abundance of food, which in turn pleased parents. Similarly, food dissatisfaction was often attributed to worrying about how to feed children. With most households reporting primary reliance on market foods, some interviewees characterized gardening, collecting, hunting, and fishing as strategies to mitigate a lack of purchased foods.

Recent literature regarding Australian Aborigines has focused on demonstrating that hybrid economies are “more than merely transitional to capitalist incorporation” [37]. Our research among the A’uwẽ population that resides in Pimentel Barbosa, Etênhiritipá, and Novo Paraíso communities, reinforces this point of view. We described earlier in this article the process by which this Indigenous group was suddenly and radically impacted in the 1970s and 1980s by economic, social, and environmental changes associated with the Brazilian government’s Xavante Project, which unsuccessfully sought to transform them into largescale commercial agriculturalists. Whereas the A’uwẽ previously depended primarily on collecting, hunting, fishing, and gardening, at the apex of the rice project they came to invest more of their time in mechanized large-scale rice production. By the 1990s, however, they returned to a more balanced mixed economy with greater emphasis on traditional food procurement accompanied by small-scale rice production for their own consumption [38]. These events are yet another example of the boom-and-bust economic cycle resulting from Brazil’s long history of beginning and then discontinuing indigenist development policies requiring large external inputs.

Other traditional peoples also engage in non-static hybrid economies with repercussions for food security. In a study of mixed-heritage coastal Caiçara communities in Brazil, food insecurity was found to be transitory because households engaged in multiple productive activities ranging from fishing and gardening to day labor and tourism, some of which are seasonal [39]. Among the Indigenous Kayapó, Zanotti [40] notes that

“*[The Kayapó] … had differing opinions on access to the market and its transformative effects on their livelihoods*. *The circulation of capital has not always been positive*, *and many Kayapó leaders and elders see too much emphasis on accumulation and individual gains as dangerous*.*”*

As shown by the author, despite all shifts in their livelihoods,

“*[…] Kayapó communities have continued to diversify their foodways and rely on local productive practices that guide harvest cycles but also intersect with individual rites of passage and community-wide events.” [40].*

We have shown that among the A’uwẽ, the hybrid economy that developed since the 1970s has proved resilient to dramatic transformations and uncertainty in the availability and characteristics of external government inputs.

If the A’uwẽ food economy of the 1970s through the 1980s was most marked by the Xavante Project, from the 1990s through the 2010s it might be considered most impacted by monetarization. With new government salaries available to Indigenous peoples in Brazil, along with the implementation of social assistance programs and other sources of monetary income, A’uwẽ households came to access market foods to greater degrees than they had in the past. Our research has shown that this shift was not uniform, and households came to rely on purchased foods to greater and lesser degrees depending on their access to monetary resources and personal preferences. At the same time, traditional food reciprocity remained an important dimension of the food economy, serving to give households with less income greater access to market foods, and leveling the access of households with greater income. Thus, the hybrid economy of the three investigated A’uwẽ communities is heterogeneous and should not be understood as the result of a unilinear or permanent transformation toward greater market participation.

The main public policies of social inclusion accessed by residents of the study communities and contributing to monetarization of the A’uwẽ food economy, which include cash transfer programs, retirement pensions, school meal programs, basic food baskets, and maternity financial assistance, should be understood as products of a specific moment in the country’s political history that may not last. As we write this article, some of these policies and programs are being debated by the federal government and may be transformed or discontinued in the future. Such changes will impact A’uwẽ communities’ and individual households’ financial profiles in unexpected ways, possibly resulting in rebalancing of the hybrid economy in accordance with available inputs, for example in favor of subsistence food production. Whereas our research among the A’uwẽ revealed a significant shift in the local hybrid economy towards market foods accompanying widespread access to social benefits and government salaries in recent decades, this has not occurred everywhere. A study in rural Amazonia has shown increased access to social benefits may not result in greater emphasis on industrialized processed foods [8]. However, the Amazon region has very different local ecological and market conditions than are experienced by A’uwẽ, including communities separated from distant market centers by expensive fluvial travel and limited local access to expensive and nondiverse retail foods.

Other factors may also affect the scales of the hybrid economy. For example, just over the course of the last few months A’uwẽ communities, along with the rest of the world, has been faced with the need to protect themselves from the Covid-19 pandemic. One community, Pimentel Barbosa, reacted with a novel solution. With the assistance of a small donation of funds to buy fuel for transportation, a large portion of the community relocated to the das Mortes River for several months up to the present, to fish and hunt for food, so as to avoid going to town to buy food. This form of self-isolation was reminiscent of the family treks the community practiced with great frequency before the Xavante Project and the reduction of their territory through federal demarcation of their lands in the 1970s and 1980s. It has resulted in greater access to traditional wild foods and reduced purchasing of market foods since the pandemic began.

It has been argued that the food security debate should not be limited to governance, but rather should emphasize place–where food actors engage–as key to effective production-consumption relations [41]. In the A’uwẽ case, for the study communities, key aspects of place include access to an undegraded cerrado landscape but very limited territorial boundaries, along with relatively easy access by road to nearby cities with banks and markets. Well-intentioned public policies of social equity and access to work that are blind to the nexus of local place and culture have functioned as cash injections into households and communities that continue to engage in reciprocity-based exchange but formerly relied more heavily on self-provisioning. They have thereby contributed to a (temporary) reorientation of the hybrid economy towards market foods and a special form of food insecurity, whereby people fear and feel hunger, but do not go hungry for very long. Nevertheless, it is difficult to presume how place and food security interact. For example, among Indigenous peoples in the middle Caquetá River region of Colombia, it was found that even favorable circumstances of intact natural ecosystems and limited access to market foods were unable to provide sufficient protection from food insecurity [42].

According to Mtika [43], food security is embedded in social and cultural relations. This view highlights the role these relations between households, especially reciprocity and redistribution, have in shaping people’s use of resources, goods, and entitlements. According to the author:

“*Rural Malawians are thus not isolated actors envisioned by the utilitarian view but social actors who constantly engage in negotiations with each other*, *sharing their entitlements*, *and thus collectively securing their food supply and diffusing burdens*. *Food security then gets compromised when burdens reach a threshold that fractures social and cultural ties thus disabling households from sharing entitlements*.*”* [43]

This account recalls A’uwẽ women’s complaints about their dissatisfaction with depleting household food resources by attending to relatives’ and neighbors’ requests for food. However, we encountered no indications of food insecurity resulting from the burdens of reciprocity being so severe that they caused social fractures that ended sharing.

To the contrary, many of our interlocuters and interviewees indicated that reciprocity and sharing were key components of their food strategies, serving to mitigate hunger and resolve food crises. This finding is similar to the results of other international studies among non-Indigenous and Indigenous populations. A study in urban Connecticut, United States, showed that increased household social capital, defined as trust, reciprocity and social networks, was associated with household food security [44]. The authors conclude that reciprocity among neighbors reduces hunger even if households have similarly limited financial or food resources. Sharing food resources, especially fish and meat, between households is also an important strategy for addressing food insecurity among Aboriginal populations in Canada [45, 46].

Our results showing less interest in traditional foods among members of younger generations is balanced by the presence of many older household members who not only like traditional foods, but actively procure them because they are tasty, healthful, and in some cases are thought to have healing powers [47]. For these older individuals, they are also an important cultural marker of A’uwẽ identity. A similar scenario was observed among Aboriginal people in Canada, with important implications for how food security is conceptualized for Indigenous peoples [48]. The author proposed that because traditional food practices are central to cultural health and survival, cultural food security should be considered an additional level within the general concept of food security that transcends the individual, household, and community levels. Central to this argument is the observation that harvesting, sharing, and consuming traditional foods involves a special relationship connecting people to the land that involves the transmission of cultural values, skills, and spirituality. According to the author, “Thus, for some Aboriginal people, the ability to access sufficient and safe traditional/country food, or food security, is integral to cultural health and survival” [48]. In Brazil, other studies have emphasized the link between Indigenous identity and food sovereignty [19].

The current situation at Pimentel Barbosa, Etênhiritipá, and Novo Paraíso communities involves a generational bifurcation. Many elders value traditional foods and wish their younger relatives would come to like them and learn how to procure them. For their part, most youth and young adults have little interest in them and prefer to rely on market foods. It is fair to say that in these communities there has not yet occurred a generalized resurgence in interest in traditional foods or amplified discourse about food sovereignty. Nevertheless, some younger people have shown great interest in collecting or hunting, having the potential to bridge the generational gap by retaining traditional ecological knowledge [47, 49]. The scenario is very different among some North American Indigenous peoples with longer colonial histories. Among multiple Indigenous ethnic groups in California and Oregon, United States, not only was lack of access to native foods prevalent and associated with high rates of food insecurity, but there was a strong demand among participants for access to desired native foods and projects to promote their availability [50]. Similarly, the Anishinaabe people in Ontario, Canada, work to protect and renew their harvesting grounds, waterways, and foodways, in ways that suggest efforts to decolonialize their food supply and promote food sovereignty [51]. Ecological research on ancient clam gardens in British Columbia, Canada, suggest how discontinued food production practices could inform and support contemporary food security strategies [52].

Identifying solutions for promoting food insecurity and sovereignty is challenging because low income and high food cost serve to limit access to healthy diets, not lack of comprehension about food requirements [53]. A’uwẽ history suggests that external factors, including development projects and social programs, can change the relationship with market and subsistence foods by altering the income/cost balance. The sedentarization of communities by such means as schools, health posts, and employment opportunities also has the potential to monetarize traditional food acquisition by transforming transportation into an extremely expensive component of excursions. Our findings are similar to those described for the Inuit in Nunavut, Canada, according to whom “food security could be gained through increased economic support for local community hunts, freezers and education programs, as well as better access to cheaper and higher quality market food” [54].

Economic relations between the A’uwẽ of the Pimentel Barbosa Indigenous Reserve and “whites” brought great transformations over the nearly eight decades since they established stable relations with the Brazilian government [20, 55]. However large these changes may be and regardless of their sphere (cultural, political, economic, and environmental), they are mediated by A’uwẽ logics strongly anchored in a complex system involving morals and aesthetics. The entry of money into communities and easy access to an immense diversity of processed foods for sale in any regional supermarket has a strong influence among young and old alike. Despite these interferences, A’uwẽ society remains faithful to its basic rites and values of its social organization, including ongoing and relevant kinship and reciprocity systems. Food is not just about “meals,” but also represents a strong identity marker that is lavishly displayed in the veritable banquets offered during wedding and initiation rites (fire roasted game meat), as well as spiritual rituals (maize bread and tubers). These ceremonies and their significance for ethnic identity and well-being serve to anchor traditional A’uwẽ foodways independently of any economic changes that may be occurring [for similar examples in Central Brazil see 40, 56]. Thus, regardless of the future direction of the country’s indigenist policies that bear on diets and food economies, there is a good chance A’uwẽ society will be sufficiently resilient within its non-static hybrid economy to maintain culturally relevant traditional ecological knowledge of cerrado foods and thereby be resilient in the face of changing circumstances of monetarization and food security.

The study communities frequently discuss in their council meetings the subject of unhealthy diets and related cardiovascular diseases, diabetes, hypertension, and excess weight. In their discourse, influential leaders have expressed their opinions that the most effective strategies to combat these problems would be actions that stimulate renewed interest in traditional food provisioning. Leaders have unsuccessfully sought to secure stable support for projects that would improve access to healthy traditional foods and encourage familiarity with the non-human environment among youth. Projects intended to promote revitalization of wild and cultivated traditional foods have tended to be ephemeral and produce limited concrete benefits. Also, there is little sign of generalized youth interest in refocusing attention on traditional foods, whether for health or heritage purposes. Nevertheless, we have observed enthusiastic interest by a minority of young people in learning and practicing traditional food procurement activities, including collecting wild plant foods among women and hunting among men. These people have the capacity to bridge future generational gaps in the transmission of traditional ecological knowledge and contribute to the cultural resilience of communities as they face unforeseen permutations in a historically boom-and-bust economy of external inputs.
